# Forced Surgeries in the Mentally Challenged Females: Ethical Consideration and a Narrative Review of Literature

**DOI:** 10.7759/cureus.26935

**Published:** 2022-07-17

**Authors:** Madhur Pradhan, Kavita Dileep, Abhijit Nair, Khalid M Al Sawafi

**Affiliations:** 1 Obstetrics and Gynecology, Ibra Hospital, Ibra, OMN; 2 Anesthesiology, Ibra Hospital, Ibra, OMN; 3 Hospital Administration, Ibra Hospital, Ibra, OMN

**Keywords:** menstrual hygiene, sterilization, hysterectomy, abortion, intellectual deficiency, mentally challenged, mentally disabled

## Abstract

Women with mental disabilities deal with menstrual problems like any other woman. The menstrual hygiene, discomfort, and sleep disturbance associated with menses make these women irritable, and their caregivers sometimes feel and decide on some unindicated surgeries like hysterectomy, sterilization, and abortion to save them from getting pregnant. While taking into consideration the burden on caregivers, complete informed choices to reach a decision are also important as these surgical procedures may do more harm than good. Early menopause happens when a hysterectomy is performed in young adults and can cause further deterioration of mental and physical health for example heart disease, and osteoporosis. Many agencies and law provide extended help and care to special children including education, and grooming which helps them achieve a life with minimum dependence. Before opting for treatment involvement of all the professionals associated with these people like psychiatrists, gynecologists, psychologists, involvement of human rights agencies, providing school education, and helping caregivers in providing care will bring some change.

## Introduction and background

The total mentally disabled population of the world is 1% [1]. Parents/caregivers of such patients, especially women with mental disabilities face the problem of menstrual hygiene, sexual abuse, and fear of unwanted pregnancies, and to combat such problems they ask the doctors to do surgeries such as hysterectomy, abortion, and sterilization. Clinicians also often deal with the dilemma of suggesting or performing surgeries like hysterectomy, abortion, or sterilization in a patient with a mental disability. The problem is not only to obtain the consent of such individuals but also to decide or do the surgery, which most of the time is not indicated. The approach to menstrual management in girls with intellectual disabilities should be the same as it is for other girls. Advice may need to be tailored according to the severity of the disability [2].

In article 5 of the United Nations Convention on the Rights of Persons with Disabilities (CRPD), it is mentioned that no one shall be subjected to torture or cruel or degrading treatment or punishment. Article 16 also gives the right to marry and find a family. But still, the disabled people who are not able to make an informed consent are victims of unnecessary and unindicated surgeries most of the time.

In this article, the issues related to consent, available treatment options, the best interest of the patient, and what the law says about the same are discussed.

## Review

Methodology

Databases like PubMed, Embase, Google Scholar, PsycINFO, Cochrane Library, and CINAHL were searched for articles published between 1986 and 2022. The keywords used for the database search were as follows: mentally disabled, mentally challenged, intellectual deficiency, abortion, hysterectomy, sterilization, and menstrual hygiene. The articles with the above-mentioned keywords were selected, downloaded, and reviewed. We excluded repetitive publications, publications dealing with male sterilization. Twenty-eight articles fulfilled the inclusion criteria.

Definition of mental disability or intellectual disability

According to the World Health Organization (WHO), intellectual disability means a significantly reduced ability to understand new or complex information and to learn and apply new skills (impaired intelligence). This results in a reduced ability to cope independently (impaired social functioning), and begins before adulthood, with a lasting effect on development [3]. According to Collins dictionary, it is a condition that limits a person's intellectual capacity, resulting directly or indirectly from injury to the brain or from abnormal neurological development [4].

History

To date, the indirect practice of eugenics and forced hysterectomies/sterilization/abortion are being done in the name of the best interest of the patient. Here is the discussion about eugenics, forced sterilization, and other surgeries [5,6]. The meaning of the word eugenics is the selection of desired heritable characteristics to improve future generations, typically humans. The term was first used in 1883 by British explorer and natural scientist Francis Galton who was influenced by Charles Darwin’s theory of natural selection. Francis Galton defined eugenics as “the science of improving stock, which is by no means confined to questions of judicious mating, but which, especially in the case of man, takes cognizance of all influences that tend in however remote a degree to give to the more suitable races or strains of blood a better chance of prevailing speedily over the less suitable than they otherwise would have had” [7].

During the 1940s, a nurse from the Immigration and Customs Enforcement (ICE) detention center in Georgia alleged that the Nazis and Americans performed numerous involuntary hysterectomies (uterus removal surgeries) on detained immigrant women [8]. In 2017, in a shocking sentence, a judge in Tennessee offered to reduce the jail sentences of convicted people who appeared before him in court provided, they volunteered for surgery to sterilize themselves. On similar lines, in 2018, an Oklahoma woman convicted of cashing a counterfeit check had her sentence reduced once she underwent sterilization. In many countries, the law is approached to get permission for performing these surgeries on mentally disabled patients. This is referred to as the doctrine of "parens patriae" [9].

Definition of parens patriae

In Latin, "parens patriae" means "parent of the people." Under the doctrine of "parens patriae," a state or court has a paternal and protective role over its citizens or other subjects under its jurisdiction. Thus, when a disabled person is not able to make a decision, the state court, family court, and Supreme Court have been approached by caregivers to seek consent for abortions, sterilization, and hysterectomy.

Review of literature

In this review, we have tried to explore problems that caregivers experience while taking care of women with mental disabilities. We have described how women with disabilities are affected because of forced surgeries done on them. We have summarized what are the laws in various countries, social services and their views, the treatment choices in children and adult females with mental disabilities who have started menstruating, abortion choices, and the need for hysterectomies or sterilization and alternative treatments [10,11].

The biggest challenge for caregivers/guardians/parents is menstrual hygiene (the care associated with that and also the fear of unwanted pregnancy due to sexual assault) To deal with these problems, caregivers often choose hysterectomy which is the removal of the womb [11], or abortion. These interventions carry the risk of anesthesia, injury to vital organs, bleeding, infection, long-term effects like incisional hernia, vault prolapse, abnormal uterine bleeding, depression, and early menopause irrespective of surgical morbidity and mortality in the mentally disabled population [12-14]. Also, one very important aspect of life is sexuality for which patients and parents need advice and the physician must be well informed and should protect the rights and autonomy of these patients. It can be carrying the pregnancy, contraception, sterilization, or hysterectomy, patients should give consent for it, and in certain situations should be decided by a multidisciplinary approach [15].

Also, the patients who are already mentally disabled have to go through the cruelty of these unindicated surgeries, pain is both physical and mental, the feeling of discrimination, and above all the threat of sexual abuse/assault still exists. To raise awareness many articles have been published arguing against and for it. Table 1 summarizes all the relevant articles related to the controversial sterilization of the intellectually impaired. Caregivers feel that it’s very difficult to control and tiring to maintain menstrual hygiene and some argued even for unwanted pregnancies, for which proxy consent by caregivers is given and doctors who performed the surgery have the view that it’s in the best interest of the patients. It is a violation of patient autonomy, as the decision to carry out the surgery is forced upon the individual which is not in the best interest of the individual but the caregivers’ interest to get rid of the burden of taking care of them. In a news article published in 1994 by Nandan G in the British Medical Journal, the author mentioned a state government in India that had given a statement that women with learning disabilities can be forced to have hysterectomies [16]. Soon after this article, the Indian Journal of Medical Ethics guidelines discussed the controversial issue of sterilization for this vulnerable group. The guidelines by the experts emphasized advocating the least injurious option for these mentally challenged cohorts [17].

**Table 1:** Summary of all the reviews, case studies, and fact sheet discussing sterilization in the intellectually impaired females

S. no.	Author(s)/year	Type of study	Conclusion
1	McKenzie et al. (2016) [1]	Systematic review	The global prevalence of intellectual disabilities may be lower than 1%.
2	Tracy et al. (2016) [2]	Review article	The starting point for women with intellectual disability management should be just the same as it would be for other women of the same age.
3	Diekema DS (2003) [6]	Review article	Less intrusive and temporary methods of contraception or control of menstruation are acceptable alternatives, and procedural safeguards have been implemented to assure a fair decision-making process.
4	Involuntary sterilization of disabled Americans (2018) [8]	Historical review	Women and girls with disabilities should be included in the evaluation and development of laws on sterilization and related matters.
5	Márquez-González et al. (2018) [10]	Systematic review	Non-therapeutic hysterectomy in intellectually disabled women should not be recommended as routine. Methods to improve menstrual hygiene should be taught.
6	Sadatmahalleh et al. (2016) [13]	Cohort study	Women with tubal ligation (TL) had more menstrual irregularity than those without TL.
7	Brown et al. (2021) [14]	Cohort study	Women with disabilities were more likely than those without disabilities to experience multiple severe maternal morbidity indicators.
8	Nandan G (1994) [16]	News article	Forcing a hysterectomy without helping in maintaining their personal hygiene cannot be justified.
9	Rana A (2020) [18]	Case report	By analyzing the risk-benefit ratio and situation analysis decisions can be made for the good of humanity.
10	Cook and Dickens (1999) [19]	Ethical and legal issues in reproductive health	Patients’ best interests must be assessed not simply by pragmatic or empirical criteria, but also by reference to common interests in respect for physical integrity and human dignity, of abled and disabled people alike.
11	Denekens et al. (1999) [20]	Review article	The decision of sterilization for a mentally challenged female should be based on a multidisciplinary consultation.
12	D'Angelo et al. (2020) [23]	Review article	Women with mental disabilities should be encouraged as much as possible to discuss sexual matters, pregnancy-related queries, and concerns with healthcare providers, especially midwives.
13	Abdulla and Rajaratnam (2019) [25]	Qualitative study	The parents/guardians should be made aware of other non or less invasive options such as the use of Depot-Medroxyprogesterone Acetate (DMPA), implants and progesterone-releasing intrauterine devices.
14	Kuppermann et al. (2004) [26]	Randomized control trial	No statistically significant difference in the intention-to-treat analysis.
15	Van der Merwe and Roux (1987) [27]	Review article	To ensure that legislation pertaining to the sterilization of the mentally retarded does not lead to abuse, inputs from the mental health professions are required.
16	Ernst and Pennesi (2020) [28]	Case study	Contraceptive counseling and planning for women with mental disabilities is challenging and complex. It is important to create awareness among obstetricians and gynecologists.
17	Pillai et al. (2010) [29]	Review article	The levonorgestrel intrauterine system (LNG IUS) provided good therapeutic benefit for the treatment of menstrual problems in adolescents with medical disorders, or physical or learning disabilities.
18	Epps et al. (1990) [30]	Comparative study	All four learners demonstrated high levels of generalized responding following on-self instruction, the effect decreased over time for two participants.
19	Richman et al. (1984) [31]	Review article	Teaching mentally retarded women to be responsible for their menstrual care needs may alleviate the embarrassment resulting from soiled clothing and odors.
20	Begun H (2008) [34]	Review article	The authors suggested using the existing knowledge base of the issues surrounding sterilization by conducting high-quality, rigorous studies that can change clinical practice to better serve not only mentally handicapped patients but also their families.
21	Fact sheet (2013) [35]	Fact sheet	The fact sheet describes significant reproductive health-related legal issues that women with disabilities.

In the medical field, we talk about non-maleficence which in simple words is “do no harm.” By doing these surgeries which are most of the time unnecessary and unindicated, the harm happens in terms of pain, infection, and risks associated with surgeries. Rana A discussed the dilemma between non-maleficence to a consequential theory where non-maleficence is “do no harm” thus sufferers’ do not be harmed [18]. While consequentialism theory considers the consequences of actions like performing a hysterectomy for intellectually disabled (ID) individuals the discomfort, irritation, restlessness, disturbed sleep, and aggressiveness associated with menstruation will subside, and the caregivers will be less burdened at the same time. But to argue for the same case here is a hypothetical theory and discussion.

Let us take an example of a 19-year-old girl for whom a progesterone intrauterine contraceptive device (IUCD) is inserted that works for five to seven years. This acts as a contraceptive and will make the female amenorrheic in another six months. If we take natural menopausal age 50 years, the female will require six times insertion of the device and will be less traumatizing to affected individuals and caregivers as well. Cook and Dickens have also discussed the role of long-acting contraception and sterilization in their article [19]. Sterilization can be opted as a method of contraception but should not be a forced decision on the mentally disabled ones. While considering long-acting reversible contraception (LARC), the medical implication of each one has to be assessed and balanced in every individual circumstance.

Denekens et al. suggested a model which is required to have a method that is ethically justified for making such decisions as sterilization and abortions [20]. The criteria suggested by them were heredity and parenting competence. They also suggested six minor criteria which comprise conception risk, intelligence quotient (IQ), age, personality, medical aspects and prognosis, and finally support and guidance for the mentally handicapped person. This model helps decide the treatment option for these individuals after considering the major and minor criteria. Before deciding on hysterectomy/sterilization considerations about schooling to teach menstrual hygiene, and other methods of treatment like levonorgestrel IUCD or etonogestrel implant might protect to some extent violations of the right to consent also.

The presence of a mental disability does not, in itself, justify either sterilization or its denial. The decision for sterilization is considered for a mentally challenged female, it is important to discuss in detail with the family members and the involved caregivers. Efforts need to be taken to preserve the autonomy of the female involved. The concept of “chronically and variably impaired autonomy” has been proposed to describe such situations [21,22]. Márquez-González et al. conducted a systematic review to determine what constitutes best practice to determine respect for autonomy and the patient's best interest [10]. They concluded that the main concern of parents was the difficulty in providing training for menstrual hygiene in low and middle-income countries. They mentioned that the government does not provide financial support or provide any training to ID women or their caretakers. This is possible only by having better lawmakers in such countries.

Is forced sterilization not against human rights?

The United Nations Convention on the Rights of Persons with Disabilities held in 2007, announced that all intellectually disabled women should be guaranteed the right to full bodily integrity. Motherhood and pregnancy were recognized as part of the rights of disabled women. However, motherhood is not always considered a right, especially by the caregivers involved [23].

Women with special needs should be given the same respect and dignity as human beings. The decision of sterilization should not be forced on them. Though their pregnancies are more complicated with diabetes, hypertension, and cesarean section (CS) higher rates but mostly it’s because of neglected pregnancy and no antenatal care. Irrespective of the level of disability the parents or caregivers are the people who will decide to carry on with the pregnancy or to force for abortion.

It is a common practice to perform hysterectomies, abortions, or sterilizations on mentally challenged girls consented by parents and approved by state agencies in government-run centers. Many countries like India are signatories to the United Nations Convention on the Rights of Persons with Disabilities 2007, which guarantees all intellectually disabled women the right to full bodily integrity [21]. Article 5 of the United Nations Universal Declaration of Human Rights states that "no one shall be subjected to torture or cruel or degrading treatment or punishment" and Article 16 confirms that "the right to marry and found a family." People with disability cannot be deprived of basic human rights. For the sake of best interests/to protect against abuse/to prevent unwanted pregnancy, these individuals are forced for these surgeries, which is against basic human rights. Few examples of such cases are discussed here. A case report submitted by Clare Dyer where a mother is asking for a hysterectomy on her daughter who is 15 years old but the mental age is 18 months, because of pain and discomfort associated with menstruation [24]. A study conducted by Abdulla et al. concluded that 50% of the population (the mothers of the intellectually disabled) were in favor of hysterectomy to combat menstrual hygiene [25].

The alternatives or the real best treatment available for these special girls and women

Menstrual hygiene, to deal with uncleanliness, sleepless nights, and discomfort is a burden on caregivers as well and therefore required a solution. The treatment options to tackle the problems generally lead to some drastic unindicated surgeries while other choices like contraception have been discussed here to prevent unintended surgeries, forced abortions, sterilization, and their adverse effects [26,27]. Contraceptive treatments at the same time make such females amenorrheic so the menstrual hygiene burden from the caregivers is reduced. The progesterone intrauterine device, implants, injections, and tablets, all after approximately six months will make a woman amenorrheic and are good choices for contraception as well. It is not easy to give such treatments as there are risk factors and some contraindications due to comorbidities in these individuals which make the choices limited. This study illustrates how to deal with the complex issues obstetrics/gynecology may encounter when counseling patients with cognitive impairment.

In a case study, a 17-year-old with a rare genetic syndrome, with mild cognitive impairment, chronic idiopathic thrombocytopenia (ITP), and migraine with aura was brought by her parents to a gynecology clinic for consultation regarding tubal ligation [28]. Contraception options were reviewed with the patient privately and were given a choice. The patient agreed to a levonorgestrel intrauterine device (IUD). The reason given by her was that she would miss less school than she would if she underwent tubal ligation. This case study discussed patient autonomy, contraceptive choices in intellectually disabled patients, contraception vs sterilization, right of the individual to reproduction. In another study conducted by Pillai et al., the levonorgestrel intrauterine system (Mirena) for the treatment of menstrual problems in adolescents with medical disorders, or physical or learning disabilities found that out of 14, 12 benefitted from its use [29].

Sterilization is not an answer for menstrual heavy bleeding and will not protect from being abused. But still, a minimally or non-invasive procedure with better effects after adjusting the risk-to-benefit ratio is needed in such cases. The other very important measures like to enforce better laws to prevent sexual assault or the need for more specialized schools where they can teach and groom women with disabilities so that they can maintain menstrual hygiene, can at least understand and report if some assault has happened or happening to them, involvement of social services to provide help to caregivers and decrease the burden from them of raising the special child alone [30,31].

Every government has a budget reserved for the disabled population which should be utilized properly. The doctor's role in such cases should be completely transparent about the surgery, its risks, complications, and benefits also as the alternative options. Education to caregivers and full discussion before performing any surgery are expected from health workers. Caregivers should be informed about the risks associated with sterilization poses a risk of abnormal uterine bleeding, while hysterectomy can cause vault prolapse, early menopause, depression, and problems associated with surgery, and anesthesia forced abortions poses a risk of infection, perforation of the uterus, and injury to other organs. Therefore, a well-thought decision with the involvement of psychiatrists, psychologists, specialized nurses, counselors, advocates, human rights agencies, social services, gynecologists, caregivers, and most importantly the individual themselves will make a difference in the treatment approach.

What does the law suggest?

Law varies from country to country [32-34]. In some countries, caregivers, doctors, advocates, and social services jointly decide on treatment. Countries like India, the USA, the United Kingdom, Columbia, and many other countries have their laws on the various procedures regarding consent. Still, in many countries including the above-mentioned countries, the law is not followed or violated. A systematic review on hysterectomy for the management of menstrual hygiene in women with intellectual disabilities focusing on standards and ethical considerations for developing countries was published by Márquez-González et al. [10]. The authors concluded that although hysterectomy as a solution to menstrual hygiene is still a controversial issue in high, middle- and low-income countries. In high-income countries, it is performed after authorization from the Court. In low- and middle-income countries, there was no active involvement of the State. Therefore, the authors suggested that in low- and middle-income countries, there is an urgent need to develop and enact policies and statutes in this area of public health and clinical practice.

In a historical review that discusses the laws and ethics surrounding the sterilization of disabled people, the historical context of the eugenics movement, and then explores current controversies regarding consent and decision-making power [35]. This fact sheet provides an overview of case law in this area as well as a brief discussion of other issues identified by legal scholars as important areas for advocacy [35]. In 1986, the Supreme Court of Canada ruled out hysterectomy for a 24-year-old, intellectually impaired lady without her consent [36]. In 1987, Braham reported a case where abortion was done based on a ruling by a British Judge who felt that in the best interests of a mentally retarded woman with a mental age of four to five years, abortion is justified [37].

## Conclusions

It is difficult to suggest simple solutions for addressing dilemmas like forced sterilization, abortions, and invasive surgeries like hysterectomies in mentally challenged females. The problems are ambiguity in the law and order, human-right issues, availability of resources, and the burden on the caregivers. There is a need for specialized laws, involvement of social services, psychologists, psychiatrists, doctors, advocates, human rights agencies, caregivers, and most importantly to consider the real best interest of the concerned individual.
